# Modification of Homologous Recombination Deficiency Score Threshold and Association with Long-Term Survival in Epithelial Ovarian Cancer

**DOI:** 10.3390/cancers13050946

**Published:** 2021-02-24

**Authors:** Jeffrey A. How, Amir A. Jazaeri, Bryan Fellman, Molly S. Daniels, Suzanna Penn, Cara Solimeno, Ying Yuan, Kathleen Schmeler, Jerry S. Lanchbury, Kirsten Timms, Karen H. Lu, Melinda S. Yates

**Affiliations:** 1 Department of Gynecologic Oncology and Reproductive Medicine, the University of Texas MD Anderson Cancer Center, Houston, TX 77030, USA; jahow@mdanderson.org (J.A.H.); aajazaeri@mdanderson.org (A.A.J.); kschmele@mdanderson.org (K.S.); khlu@mdanderson.org (K.H.L.); 2 Department of Biostatistics, the University of Texas MD Anderson Cancer Center, Houston, TX 77030, USA; BMFellman@mdanderson.org (B.F.); yyuan@mdanderson.org (Y.Y.); 3 Department of Clinical Cancer Genetics, the University of Texas MD Anderson Cancer Center, Houston, TX 77030, USA; msdaniel@mdanderson.org; 4 Myriad Genetics, Salt Lake City, UT 84108, USA; schau@myriad.com (S.P.); csolimen@myriad.com (C.S.); jlanchbu@myriad.com (J.S.L.); ktimms@myriad.com (K.T.)

**Keywords:** epithelial ovarian cancer, homologous recombination deficiency, homologous recombination deficiency score, microsatellite instability, tumor mutational burden, survival

## Abstract

**Simple Summary:**

Knowledge of ovarian cancer molecular characteristics is increasingly crucial to individualizing and optimizing treatment strategies for this heterogeneous disease. Molecular features such as germline or somatic mutations, homologous recombination deficiency (HRD) status, microsatellite instability, and tumor mutational burden are associated with increased susceptibility to poly-ADP ribose polymerase inhibitors (PARPi) or immunotherapy. Our aim was to characterize these molecular features among ovarian cancer patients and determine their association with survival. Two different HRD score thresholds were evaluated: one currently used in clinics (≥42) and another proposed as a new threshold (≥33). An HRD score ≥33 was associated with improved overall survival in ovarian cancer. As HRD assays are increasingly used for treatment planning, future studies should evaluate an HRD score threshold of ≥33 compared to the currently used threshold of ≥42 for PARPi use.

**Abstract:**

New therapies, such as poly-ADP ribose polymerase inhibitors (PARPi), and immunotherapy treatments have generated great interest in enhancing individualized molecular profiling of epithelial ovarian cancer (EOC) to improve management of the disease. In EOC patients, putative biomarkers for homologous recombination deficiency (HRD), microsatellite instability (MSI), and tumor mutational burden (TMB) were characterized and correlated with survival outcomes. A series of 300 consecutive EOC patients were enrolled. Patients underwent neoadjuvant chemotherapy (*n* = 172) or primary cytoreductive surgery (*n* = 128). Molecular profiling and survival analyses were restricted to the primary cytoreductive surgery cohort due to tissue availability. All patients underwent germline testing for HRD- and MSI-related gene mutations. When sufficient tissue was available, screening for somatic BRCA1/2 mutations, BRCA1 promoter methylation, HRD score (a measure of genomic instability), MSI, and TMB testing were performed. HRD score ≥33 was associated with improved overall survival on multivariable analysis. In the era of biomarker-driven clinical care, HRD score ≥33 may be a useful adjunctive prognostic tool and should be evaluated in future studies to predict PARPi benefits.

## 1. Introduction

Epithelial ovarian cancer (EOC) is the most deadly gynecologic malignancy, responsible for an estimated 13,940 deaths annually in the United States [1]. Historically, EOC has been clinically managed as one homogeneous entity with a combination of surgical cytoreduction and platinum-taxane chemotherapy [2,3,4]. However, histologic subtypes of EOC demonstrate significant biologic and genetic differences that impact their susceptibility to cytotoxic chemotherapy and targeted agents [2]. The most common EOC histologic subtype is high-grade serous (HGS) carcinoma (70% of EOC cases), with the remainder being low-grade serous, clear cell, endometrioid, and mucinous [5]. Additionally, in profiling the molecular landscape of ovarian tumors, the Cancer Genome Atlas (TCGA) identified distinct molecular features between histologic subtypes. For example, HGS tumors had near universal TP53 gene mutations (96%), with half containing abnormalities in the homologous recombination (HR) pathway [6]. Focusing on unique molecular features of EOC can provide the foundation to advance clinical management beyond the historical one-size-fits-all approach and improve long-term clinical outcomes.

Exploiting defects in the HR pathway has transformed clinical management of EOC and has placed emphasis on the development of reliable molecular assays to identify the presence of HR defects. The HR pathway is a set of critical DNA damage response mechanisms that protect genomic stability through high-fidelity repair of double-stranded DNA breaks [5,7,8]. Among a number of HR pathway protein factors, BRCA1 and BRCA2 proteins are critical to the integrity of the HR repair response. BRCA1 is a versatile protein that complexes with a number of proteins in the BRCA1-associated genome surveillance complex in order to link the detection of double-stranded DNA breaks and DNA damage repair effectors [5,8,9]. The main function of the BRCA2 protein is recruitment of RAD51 to regions of double-stranded breaks for HR repair [5,8,10]. With defective HR pathway proteins, cells with homologous recombination deficiency (HRD) are more susceptible to the intra- and interstrand crosslinking action of platinum agents [11,12]. Furthermore, HRD has become an attractive target for poly-ADP ribose polymerase inhibitors (PARPi) due to mechanisms of synthetic lethality and/or PARP trapping [5,13,14,15]. Given the crucial involvement of BRCA1 and BRCA2 proteins in the HR pathway, PARPi treatment in patients with BRCA1/2 mutations has significantly improved progression-free survival (PFS), thereby dramatically changing the landscape for EOC management [16,17,18,19]. Given this clinical benefit, expanding the scope of PARPi use through the identification of other biomarkers of HRD has been a particular focus of research [5,15]. In addition to germline and somatic BRCA1/2 mutations, other HRD markers include BRCA1 promoter methylation and other HR-related gene mutations (e.g., ATM, BARD1, BRIP1, CHEK2, NBN, PALB2, RAD51C, and RAD51D) [5,20]. Another assay that has garnered strong interest is the combined homologous recombination deficiency score (HRD score) [19,21,22,23,24,25]. HRD score is an unweighted sum of three independent DNA-based measures of genomic instability (loss of heterozygosity, telomeric allelic imbalance, and large-scale transitions) in the tumor [19,21,22,23,24,25]. A high HRD score (≥42) has been shown to be predictive of clinical benefit with PARPi therapy, independent of BRCA1/2 status [26,27].

Immunotherapy also holds promise to revolutionize management of EOC and improve clinical outcomes, but again, requires robust and clinically feasible molecular assays to define key features and predict treatment response. Tumor molecular features, such as microsatellite instability (MSI), increase tumor immunogenicity and are strongly predictive of response to immune checkpoint inhibitors [28]. Microsatellites are repeated segments of DNA that are prone to DNA replication errors and these errors are typically corrected by DNA mismatch repair (MMR) proteins [29]. Nonfunctional or absent MMR proteins result in a substantial increase in mutations in the microsatellite regions, a condition called microsatellite instability [29]. Given this genetic hypermutable state, MSI or MMR-deficient tumors produce a greater neoantigen load and elicit a stronger antitumor immune response compared to microsatellite stable or MMR-proficient tumors [29]. Marabelle et al. reported improved objective response rates among patients with recurrent MSI or MMR-deficient EOC tumors treated with pembrolizumab [30,31]. Given this clinical benefit, pembrolizumab was approved for use in MSI or MMR-deficient solid tumors in 2017, signifying the first tissue-agnostic indication for a drug. Furthermore, high tumor mutational burden (TMB), a measure of the number of gene mutations within the tumor, has been cited as a predictive biomarker for response to immune checkpoint inhibitors [32].

Given the clinical importance of molecular profiling for HRD, MSI, and TMB, we developed a consecutive series of cases of EOC to conduct both molecular characterization and collect extensive clinical follow-up data. With this powerful cohort and molecular assays, we evaluated the association of these biomarkers and other clinical prognostic factors with survival outcomes.

## 2. Materials and Methods

### 2.1. Patient Population

Under a protocol approved by the institutional review board, a consecutive series of 300 EOC patients undergoing frontline treatment (01/2010 to 12/2013) were prospectively enrolled for tumor and germline BRCA1/2 characterization and additional tumor analyses. Cases included patients at MD Anderson Cancer Center and Lyndon B. Johnson General Hospital (treatment provided by the same gynecologic oncology attending faculty at both sites). The study inclusion criteria were the following: suspected or biopsy-proven epithelial ovarian, fallopian tube, or primary peritoneal cancer with adequate tumor specimen collection (>0.5 g) and/or willing to have blood sample collection for molecular testing. Exclusion criteria included patients presenting for treatment of recurrent disease or mucinous or borderline histologic subtypes. Tumors were classified as HGS histology if they contained a HGS component—this classification consisted of tumors with uniform HGS histology or mixed histology (e.g., endometrioid and HGS components). Chemotherapeutic regimens for patients undergoing primary cytoreductive surgery (PCS) or neoadjuvant chemotherapy (NACT) consisted of platinum (carboplatin or cisplatin) and taxane agents (paclitaxel or docetaxel). Due to limited tissue availability of specimens from pretreatment tumor biopsies for patients undergoing NACT, molecular characterization of tumor samples focused on patients who underwent PCS. Due to this limitation, while overall cohort characteristics are described (Table 1), the scope of the molecular evaluation was restricted to the PCS subpopulation.

### 2.2. Blood Sample Collection and Molecular Testing

Blood samples were collected perioperatively to perform germline testing for BRCA1/2 mutations and other genes involved in the HR pathway, including: ATM, BARD1, BRIP1, CHEK2, NBN, PALB2, RAD51C, and RAD51D. Other genes included in the genomic panel were the following: APC, BMPR1A, CDH1, CDK4, CDKN2A, EPCAM, MLH1, MSH2, MSH6, MUTYH, PMS2, PTEN, SMAD4, STK11, and TP53. Germline mutations that were deleterious or suspected deleterious were considered to be clinically significant mutations. Genomic DNA was extracted from blood samples and the assays were performed using next generation sequencing (NGS) and large rearrangement detection analysis, as previously described by Judkins et al. [33].

### 2.3. Tumor Testing

Molecular testing was retrospectively performed on snap frozen tumor samples that were collected prior to initiation of chemotherapeutic treatment. If frozen tumor tissue was unavailable, formalin-fixed paraffin embedded (FFPE) tissue was evaluated. BRCA1 promoter methylation levels were quantified using DNA methylation PCR arrays as previously described [21]. BRCA1/2 mutation screening was performed using custom hybridization enrichment and NGS on DNA from FFPE samples as described previously [34]. HRD score was determined by custom hybridization sequencing assay and was defined as the cumulative sum of whole-genome tumor loss of heterozygosity, telomeric allelic imbalance, and large-scale state transition, as described previously [24,25]. Tumor samples with HRD scores ≥42 were considered to be HR deficient. Given that recent studies have suggested that lowering the HRD score cut-off from 42 to 33 may improve sensitivity to detecting a response to PARPi, HRD characterization and univariable/multivariable analyses were also performed with HR deficiency, as defined by HRD score threshold ≥33 [35,36,37,38]. TMB was evaluated using a single nucleotide polymorphism-based resequencing assay, as previously described and validated [39]. MSI testing was performed as previously described [40].

### 2.4. Clinical Data Collection

Clinical data were extracted from electronic medical records, including age, race/ethnicity, center, stage, tumor grade, histology, residual disease volume following surgery, chemotherapy cycles, recurrence, and death. Study data were collected and managed using REDCap (Research Electronic Data Capture) electronic data capture tools hosted at MD Anderson [41].

### 2.5. Statistical Analysis

Descriptive statistics were used to summarize the demographic and clinical characteristics of the study population. Overall survival (OS) was measured from date of surgery to date of death or last follow-up. Patients alive were censored at date of last follow-up. Median follow-up was defined as the median time from date of initial treatment (PCS or NACT) to date of last follow-up among those who were alive at the last follow-up visit. PFS was measured from date of surgery to the date of earliest recurrence, progression, or death. Patients were censored on the date of last evaluation for recurrence or progressive disease. The product limit estimator of Kaplan and Meier was used to estimate OS and PFS and modeled via Cox proportional hazards regression as a function of prognostic factors to estimate the hazard ratio with 95% confidence intervals. Separate multivariable models were constructed adjusting for age, stage, histology, and debulking status; low-grade histologic subtypes were excluded from these multivariable models. Exploratory subset analyses were conducted separately within the HGS subset. All statistical analyses were performed using Stata/MP v16.0 (College Station, TX, USA).

## 3. Results

### 3.1. Patient Population

**Figure 1:**
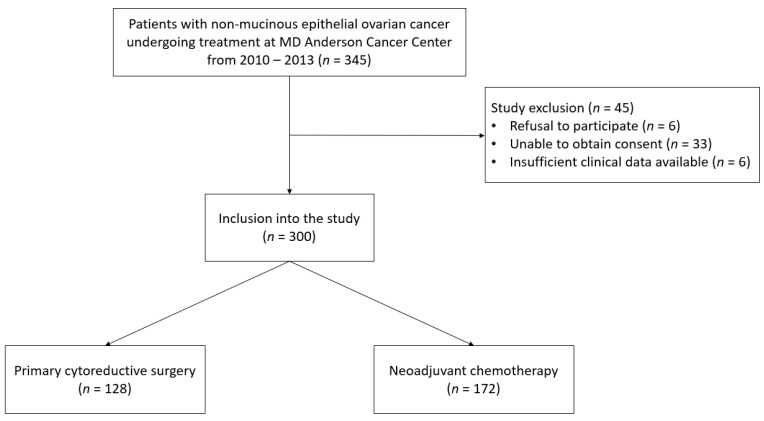
Flow diagram of patient population.

A total of 300 EOC patients were enrolled meeting study criteria (Figure 1). Of the 300 patients, 128 patients (42.7%) underwent PCS and the remaining 172 patients (57.3%) underwent NACT followed by interval cytoreductive surgery (Figure 1).

The study period predated PARPi approvals for frontline maintenance therapy and thus none of the patients received this treatment. Demographic and clinical characteristics of the full cohort of 300 patients are described in Table 1. In the PCS cohort, the median age was 61 years (range 24–83), with a median follow-up of 79.1 months (range 18.6–114.9). The majority of patients had advanced stage disease (79.8%) and HGS histology (71.9%). Information regarding residual tumor volume following PCS was available for 112 patients. Optimal cytoreduction occurred in 103 of 112 patients (92%) with 70 achieving no residual disease (R0), 18 having residual disease ≤1 cm as the largest tumor diameter, and 15 cases were unspecified. There were differences in stages between the PCS and NACT cohorts (*p* < 0.001). The NACT cohort had a greater proportion of stage III/IV patients (80.1% vs. 97.7%). Additionally, there were differences in histology subtypes between the PCS and NACT cohorts (*p* < 0.001). The NACT cohort had a greater proportion of HGS histology (71.9% vs. 90.1%, respectively) and a smaller proportion of endometrioid (7.0% vs. 0.6%) and clear cell (7.0% vs. 1.2%) histology. Molecular characterization focused on the PCS cohort due to the general availability of abundant pretreatment tumor tissue.

### 3.2. Characterization of HRD, MSI, and TMB

**Figure 2:**
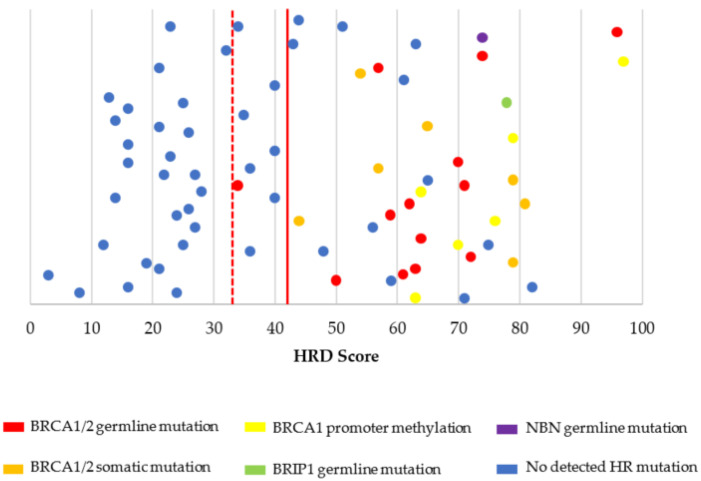
HRD score values and their associated HR defects in high-grade serous histology (*n* = 74). HR = homologous recombination. HRD = homologous recombination deficiency. HRD score cut-off threshold was defined as ≥42 (red solid line). HRD score values above this threshold line were considered high HRD scores (HR deficient tumors) and values below the threshold line were considered low HRD scores (HR proficient tumors). An HRD score cut-off threshold ≥33 (red dotted line) was also evaluated. Please note that values on the *y*-axis have been scattered to allow easier visualization of the HRD score datapoints. There is no variable on the *y*-axis.

**Figure 3:**
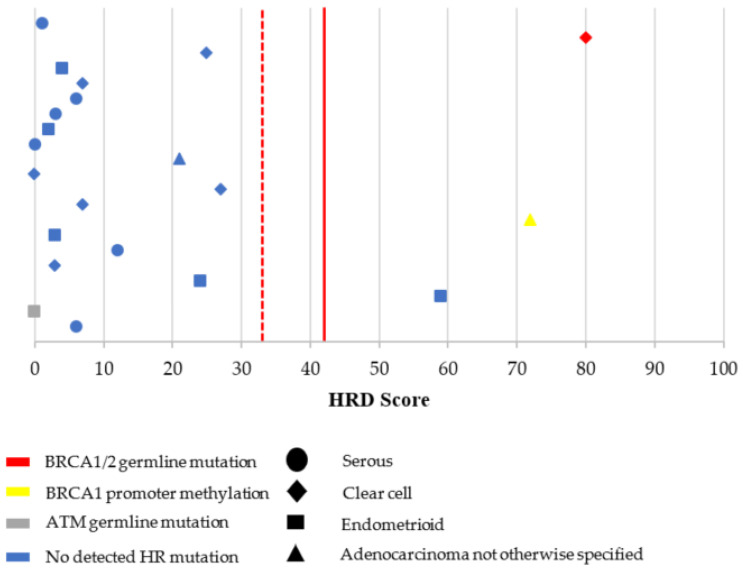
HRD score values and their associated HR defects in non-high-grade serous histology (*n* = 21). HR = homologous recombination. HRD = homologous recombination deficiency. HRD score cut-off threshold was defined as ≥42 (red solid line). HRD score values above this threshold line were considered high HRD scores (HR deficient tumors) and values below the threshold line were considered low HRD scores (HR proficient tumors). An HRD score cut-off threshold ≥33 (red dotted line) was also evaluated. Please note that values on the *y*-axis have been scattered to allow easier visualization of the HRD score datapoints. There is no variable on the *y*-axis.

Molecular testing characteristics for the overall PCS cohort are shown in Table 2 and Figure 2 and Figure 3. Among patients who underwent PCS with completed BRCA1/2 germline testing, there were 17 patients (13.9%) with germline BRCA1/2 mutations (10 BRCA1 and 7 BRCA2). Suspected deleterious or deleterious germline mutations in other non-BRCA1/2 mutations in the HR pathway were present in five of 85 patients (5.9%) and included BRIP1 (*n* = 2), ATM (*n* = 2), and NBN (*n* = 1). Molecular testing of tumor tissue identified an additional five and three patients with somatic BRCA1 and BRCA2 mutations, respectively. BRCA1 promoter methylation was present in seven patients (7.5%). The vast majority of ovarian tumors had microsatellite stability (96.4%) and low TMB (98.9%) (Appendix A Table A1). Microsatellite instability was identified in one case, which included clear cell histology and high TMB (30.4 mutations/megabase). MSI testing failed for three patients (2.7%) (Table A1). HRD score testing was attempted in 108 patients with completed testing in 95 (88.0%). Failed testing was most commonly due to low tumor cellularity in the testing specimen. Details of detected mutations associated with HRD are shown in Figure 2 and Figure 3.

Among HGS cases with complete HRD characterization and an HRD score ≥42, 9 of 17 patients (52.9%) had an associated cause for HRD (four germline BRCA1, two germline BRCA2, one somatic BRCA2, and two BRCA1 promoter methylation). When evaluating an HRD score ≥33, a total of 20 patients had complete HRD characterization and had an HRD score ≥33; however, no specific HR defects were determined in these three additional cases from our panel of molecular features.

Examining the non-HGS histology cases, there were 3 out of 21 (14.3%) patients with an HRD score ≥42 (Figure 3). These cases included one case with clear cell histology (HRD score of 80 and associated with a germline BRCA1 mutation), one case with adenocarcinoma not otherwise specified (HRD score of 72 and BRCA1 promoter methylation), and one case with endometrioid histology (HRD score of 59, but somatic BRCA1/2 and germline HR testing was not performed). There were no additional patients with non-HGS histology who met the criteria for HRD when the HRD score threshold was lowered to 33.

Overall, among all patients with successful HRD score testing (*n* = 95), there were 50 patients who met the criteria of HRD based on HRD score (≥33). Among the 50 patients with negative germline and somatic BRCA1/2 results (34 HGS and 16 non-HGS) and successful HRD score testing, there were 20 with HRD scores ≥33.

### 3.3. Survival

Univariable analyses for OS and PFS for patients undergoing PCS are described in Table A2 and Table A3, respectively. Overall, the median OS and PFS was 84.3 (95% CI 66.2–not reached) and 23.6 (17.3–36.1) months, respectively. As anticipated, younger age (<65 years), early stage, and optimal cytoreduction were associated with improved OS and PFS (Table A2 and Table A3). Any BRCA1/2 mutation was associated with improved OS (HR 0.36, 95% CI 0.17–0.77; *p* = 0.008) and PFS (HR 0.56, 95% CI 0.33–0.98; *p* = 0.041). OS and PFS univariable analyses based on HRD score threshold are described in Table 3 and Table 4, respectively.

**Figure 4:**
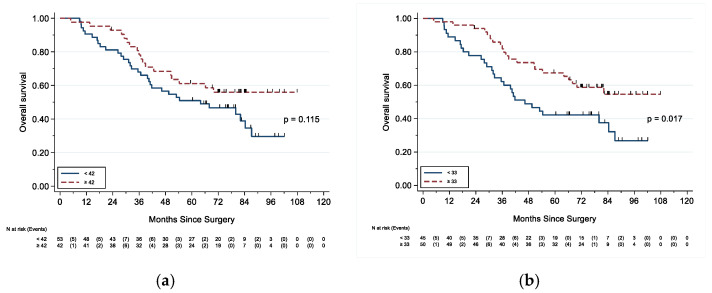
Overall survival based on HRD score threshold. (**a**) Overall survival based on HRD score threshold of 42; (**b**) overall survival based on HRD score threshold of 33.

Although an HRD score ≥42 was not associated with differences in OS nor PFS, an HRD score ≥33 was associated with improved OS (HR 0.51, 95% CI 0.29–0.90; *p* = 0.019) (Table 3 and Figure 4).

Subgroup univariable analyses for patients with HGS tumors are also shown in Table A2, Table A3, Table 3 and Table 4. Again as expected, patients with HGS tumors who were younger in age, in an early stage, had optimal cytoreduction, or had any BRCA1/2 mutation had improved OS and PFS (Table A2 and Table A3). An HRD score ≥42 was associated with improved OS (HR 0.48, 95% CI 0.26–0.90; *p* = 0.021) and PFS (HR 0.57, 95% CI 0.34–0.95; *p* = 0.033) among HGS tumors (Table 3 and Table 4). Similarly, an HRD score ≥33 was also associated with improved OS (HR 0.31, 95% CI 0.17–0.58; *p* < 0.001) and PFS (HR 0.46, 95% CI 0.27–0.77; *p* = 0.003) (Table 3 and Table 4).

Results of the multivariable analyses are shown in Table 5. Excluding low-grade tumors, the multivariable model adjusted for age, stage, histology, and debulking status. An HRD score ≥33 (HR 0.43, 95% CI 0.23–0.81; *p* = 0.009) or any HR defects (with HRD score ≥33) (HR 0.45, 95% CI 0.23–0.89; *p* = 0.022) were significantly associated with improved OS when adjusting for age, stage, histology, and debulking status. For PFS, any BRCA1/2 mutation was associated with improved outcome (HR 0.47, 95% CI 0.24–0.92; *p* = 0.028).

## 4. Discussion

With growing clinical need and interest in molecular tumor assays in the management of EOC, we sought to characterize HRD biomarkers, MSI, and TMB and determine their associations with survival outcomes. Given the potential clinical impact of the HRD score assay, evaluation in multiple study populations is essential. We evaluated the impact of lowering the HRD score threshold from 42 to 33, given that retrospective studies have suggested that such a cutoff may improve sensitivity in detecting responses and benefits to platinum-containing agents and/or PARPi [35,36,37,38]. Both HRD score thresholds of 42 or 33 were associated with improved OS and PFS among patients with HGS tumors in univariable analyses. However, compared to the previously used threshold of 42, the lower threshold of 33 remained significantly associated with improved OS among all patients in univariable and multivariable analyses. Additionally, in multivariable analyses, improved PFS for those with HRD scores ≥33 approached statistical significance.

After lowering the HRD score threshold to 33, there were an additional 8 patients (making a total of 50 patients) who met the criteria of HRD based on HRD score alone, out of a total of 95 with completed HRD score testing. Among the 95 patients with complete HRD score testing, there were also 50 with negative germline and somatic BRCA1/2 results (34 HGS and 16 non-HGS), with 20 having high HRD scores. Thus, based on the findings in this study, HRD score testing could help identify a substantial number of patients with HRD beyond patients that would be identified with BRCA1/2 testing alone. Furthermore, among those with HRD testing, there was interestingly only one patient with an HR defect who did not have a high HRD score ≥33 (a patient with endometrioid histology, ATM germline mutation, and an HRD score of 0). Although ATM plays an early role in the HR pathway, there is evidence that ATM-deficient cells may still have overall functional HR pathways (albeit delayed kinetics) with sensitivity to PARPi because ATM is postulated to also regulate response to double-strand DNA breaks at multiple levels and inhibit error prone nonhomologous end joining [42,43]. This mechanism may explain the low HRD score in the aforementioned patient with a germline ATM mutation.

Given the growing importance of immunotherapy in solid tumors and the need to establish biomarkers to predict responses, we evaluated MSI and TMB in the EOC population undergoing PCS. In our study, we observed only one MSI tumor (clear cell histology) out of 107 (0.9%) EOC tumors with successful MSI testing. This finding is consistent with the other studies that have reported an MSI prevalence of approximately 1–2% [44,45]. A meta-analysis and systematic review by Pal and colleagues highlighted the wide range in the prevalence of MSI in 977 EOC patients in 18 studies ranging from 0% to 36.7% [46] and a pooled frequency of 12% (95% CI 8–17%), but the variations in reported prevalence may be related to interstudy differences, including study population heterogeneity, histologic subtypes evaluated, or detection methods [45,46]. Additionally, we observed TMB to be low in nearly all our patients except for the same patient with an MSI tumor. Other studies have reported TMB to be lower in EOC compared to other tumors but that it may be increased in tumors in the presence of HR defects [47,48]. It is difficult to comment on the relevance of MSI and TMB status on prognosis given that the overwhelming majority of tumors did not demonstrate MSI and had low TMB. In the literature, there have been mixed results regarding MSI and TMB for EOC patients with MSI tumors [49,50]. Future studies should investigate MSI and TMB in the non-HGS EOC population.

As expected, factors such as younger age, early stage, optimal cytoreduction, or presence of BRCA1/2 mutations were associated with improved OS and PFS (especially for HGS patients) and these prognosticators have been reported in the literature [51,52]. We did not observe any association between BRCA1 promoter methylation and survival and it is difficult to assess the impact of BRCA1 promoter methylation in this study given the small number of cases with methylation (*n* = 7). Unlike germline or somatic BRCA1/2 mutations, BRCA1 promoter methylation as a prognosticator has been reported with mixed results [53,54,55]. Similar to BRCA1 promoter methylation in this study, there were few cases of germline non-BRCA1/2 HR mutations and this likely impacted the ability to observe a favorable association with survival outcomes. This result is in contrast with the results of molecular analyses of samples from patients enrolled in the larger GOG 218 trial (*n* = 307), where the investigators observed non-BRCA1/2 HR-related mutations to be a favorable prognosticator [20].

The utilization of HRD score testing in clinical trials evaluating PARPi therapy has highlighted the assay’s potential clinical benefit to EOC patients. In a phase III, double-blind randomized control trial (PRIMA), patients with HRD score ≥42 had improved PFS when treated with niraparib compared to placebo (HR 0.50, 95% CI 0.31–0.83) in the frontline setting [56]. In a phase II, single-arm QUADRA trial, Moore and colleagues observed an objective response rate of 26% to niraparib for recurrent, platinum-sensitive EOC patients with HRD scores ≥42 (compared to 4% in the HR proficient or unknown group) on subgroup analysis [27]. The results of the QUADRA trial led to FDA approval of niraparib monotherapy for patients with platinum-sensitive tumors and a high HRD scores in the recurrent setting. Additionally, a phase III PAOLA-1 trial investigated the use of bevacizumab (anti-VEGF monoclonal antibody) and olaparib maintenance compared to placebo/bevacizumab maintenance in advanced stage EOC in the frontline setting [26]. In a subgroup analysis of patients with an HRD score ≥42, the median PFS was improved in the olaparib/bevacizumab arm compared to the placebo/bevacizumab arm (28.1 vs. 16.6 months; HR 0.31, 95% CI 0.20–0.47) [26]. Based on these results, the addition of olaparib to bevacizumab maintenance in the frontline setting received FDA approval in May 2020 for patients with advanced EOC and HR deficient tumors that are responsive to platinum-based chemotherapy and bevacizumab. In a phase III VELIA trial, patients with advanced HGS ovarian cancer in the frontline setting were placed in three arms: chemotherapy with placebo and placebo maintenance (control), chemotherapy with velaparib followed by placebo maintenance (velaparib-combination only), and chemotherapy with velaparib followed by velaparib maintenance (velaparib-throughout) [36]. The trial designated HRD scores ≥33 as HR deficiency, given that retrospective analyses had demonstrated a subset of patients with HRD scores below 42 who derived some PFS benefit when treated with PARPi [35,37,38]. The VELIA investigators demonstrated an HRD score ≥33 was associated with improved PFS (HR 0.80, 95% CI 0.64–0.997) when treated with velaparib-throughout compared to placebo [36]. These aforementioned clinical trials highlighted the importance of HRD score testing as a tool to identify patients likely to benefit from PARPi therapy. Biomarkers such as germline/somatic BRCA1/2, germline non-BRCA1/2 mutations, and BRCA1 promoter methylation may not fully capture defects in the HR pathway. Thus, genomic signatures captured through HRD assays can potentially expand the benefit of PARPi to more EOC patients.

One of the strengths of this study was the follow-up period for the study population of 79.1 months (range 18.6–114.9 months). A long follow-up period improves the ability to detect differences in OS based on biomarker status. Furthermore, this study evaluated multiple biomarker surrogates for HRD in order to evaluate evidence of HRD. Additionally, this cohort had a larger than usual representation of Hispanic patients (13.3% compared to typically 4%) [20,57]. This study has several limitations. Molecular analyses could not be uniformly performed on all cases. When performing molecular analyses, blood or tissue samples were not always sufficient to complete germline non-BRCA1/2 HR-related mutation, BRCA1 promoter methylation, or HRD score testing. In addition, because only pretreatment core biopsies were available for patients in the NACT cohort, these specimens were reserved for clinical care and this research study focused exclusively on the PCS cohort. Furthermore, PCS and NACT populations may vary across institutions due to different practice patterns, which may influence the generalizability of these findings. Additionally, given the longitudinal nature of the study, long-term survival outcomes may also be influenced by differences in treatment regimens following frontline therapy. Furthermore, as discussed earlier, the clinical impact of studied prognosticators may also be modified in the current landscape of greater PARPi use. In addition, there were low numbers of endometrioid, clear cell, and low-grade serous tumors for HRD characterization. This representation was expected given that HGS tumors represent the great majority of EOC. Lastly, while the HRD ≥42 group did not show a statistically significant association with overall survival in the Kaplan–Meier analysis or multivariable models, it is possible that evaluation in a larger cohort of cases would have greater statistical power to determine an association. Nevertheless, it is important to note that the hazard ratios for the HRD ≥33 group showed consistently significant HRD effects compared to the HRD ≥42 group. In the multivariable models, evaluation of these two different cutoffs again showed a significant HRD effect using the HRD ≥33 cutoff but not when using the HRD ≥42 cutoff (Table 5).

## 5. Conclusions

In conclusion, EOC management is becoming increasingly individualized as molecular features guide treatment stratification. The HRD score assay provides a valuable adjunctive tool to capture evidence of HRD that may be missed if only germline HR and somatic BRCA1/2 testing are performed. An HRD score threshold ≥33, compared to the currently used threshold of ≥42, has a strong association with improved overall survival. This new threshold may serve to better prognosticate patients with EOC and has the potential to expand the number of candidates who could receive PARPi as an alternative treatment option. Future studies are needed to compare the predictive ability of both these thresholds on PARPi therapeutic benefit.

## Figures and Tables

**Table 1:** Demographic and clinical characteristics.

Clinicopathologic Characteristic	PCS (*n* = 128)		NACT (*n* = 172)		*p*
	N	%	N	%	
Age					0.625
Median (range)	61 (24–83)		62 (25–95)		
Race/ethnicity					0.500
White	97	75.8	123	72.4	
African American/Black	5	3.9	13	7.6	
Asian	9	7.0	15	8.8	
Hispanic	17	13.3	19	11.2	
Unknown	0	0	2	N/A	
Disease site					0.022
Fallopian tube	10	7.9	6	3.5	
Ovary	95	75.4	117	68	
Peritoneum	21	16.7	49	28.5	
Unknown	2	N/A	0	0	
Stage					<0.001
In Situ	1	0.8	0	0.0	
I	11	9.2	0	0.0	
II	13	10.8	1	0.7	
III	82	68.3	72	53.3	
IV	13	10.8	60	44.4	
Advanced	0	0.0	2	1.5	
Unknown/unstaged	8	N/A	37	N/A	
Histology					<0.001
Serous					
High grade	92	71.9	155	90.1	
Low grade	16	12.5	12	6.9	
Endometrioid					
High grade	8	6.2	1	0.6	
Low grade	1	0.8	0	0.0	
Clear cell	9	7.0	2	1.2	
Adenocarcinoma NOS	2	1.6	2	1.2	
Cytoreductive surgery					0.356
Optimal	103	92.0	139	88.5	
R0	70	68.0	90	57.3	
≤1 cm	18	16.1	29	18.5	
Not specified	15	13.4	20	12.7	
Suboptimal (>1 cm)	9	8.0	18	11.5	
Unknown	16	N/A	15	N/A	
Follow-up (months)					0.870
Alive at time of analysis	68	53.1%	45	26.2%	
Median (range)	79.1 (18.6–114.9)		79.2 (9.3–105.4)		

N/A = not applicable. NACT = neoadjuvant chemotherapy. NOS = not otherwise specified. PCS = primary cytoreductive surgery. R0 = no macroscopic residual tumor. Note: cases with “unknown” characteristics are not included in percentages shown. N/A = not applicable. NACT = neoadjuvant chemotherapy. NOS = not otherwise specified. PCS = primary cytoreductive surgery. R0 = no macroscopic residual tumor.

**Table 2:** Molecular testing characteristics in patients undergoing PCS.

Germline Testing	*n* (%)
gBRCA1/2 status (*n* = 122)	
gBRCA1/2 negative	105 (86.1%)
gBRCA1 mutation ^1^	10 (8.2%)
gBRCA2 mutation ^1^	7 (5.7%)
Other HR gene mutations (*n* = 85)	
BRIP1	2 (2.4%)
ATM	2 (2.4%)
NBN	1 (1.2%)
MMR gene mutations ^2^ (*n* = 85)	0 (0%)
Other germline mutations (*n* = 85)	
MUTYH	2 (2.4%)
Tumor testing	*n* (%)
sBRCA1/2 status (*n* = 68)	
sBRCA1/2 negative	59 (86.7%)
sBRCA1 mutation	5 (7.4%)
sBRCA2 mutation	3 (4.4%)
BRCA1 promoter methylation (*n* = 93)	
≥15%	7 (7.5%)
<15%	86 (92.5%)
HRD score (*n* = 95)	
≥42	42 (44.2%)
≥33	50 (52.6%)

^1^ 16 of 16 patients with a germline BRCA1/2 mutation (10 BRCA1 and 6 BRCA2) with both germline and tumor BRCA1/2 testing results confirmed their respective germline mutations in the tumor. One patient with a germline BRCA2 mutation did not undergo somatic BRCA1/2 testing. ^2^ Includes MLH1, MSH2, MSH6, PMS2, EPCAM. gBRCA1/2 = germline BRCA1/2 mutation. HR = homologous recombination. HRD = homologous recombination deficiency. MMR = mismatch repair. sBRCA1/2 = somatic BRCA1/2 mutation. PCS = primary cytoreductive surgery.

**Table 3:** Overall survival in patients undergoing PCS by HRD score.

	Overall (*n* = 128)			HGS (*n* = 92)		
Characteristic	N	Hazard Ratio (95% CI)	*p*	N	Hazard Ratio (95% CI)	*p*
HRD score						
<42	53	Ref		35	Ref	
≥42	42	0.63 (0.35–1.12)	0.118	39	0.48 (0.26–0.90)	0.021
HRD score						
<33	45	Ref		27	Ref	
≥33	50	0.51 (0.29–0.90)	0.019	47	0.31 (0.17–0.58)	<0.001
Any HRD (HRD score ≥42) ^1^						
No	42	Ref		26	Ref	
Yes	49	0.59 (0.33–1.05)	0.074	45	0.45 (0.24–0.85)	0.014
Any HRD (HRD score ≥33) ^2^						
No	36	Ref		20	Ref	
Yes	56	0.49 (0.27–0.87)	0.014	52	0.29 (0.15–0.55)	<0.001

HGS = high-grade serous. HRD = homologous recombination deficiency. PCS = primary cytoreductive surgery. ^1^ Any HRD = germline or somatic BRCA1/2 mutation, germline non-BRCA1/2 homologous recombination deficiency mutation, BRCA1 promoter methylation (≥15), and/or HRD score ≥42). ^2^ Any HRD = germline or somatic BRCA1/2 mutation, germline non-BRCA1/2 homologous recombination deficiency mutation, BRCA1 promoter methylation (≥15), and/or HRD score ≥33).

**Table 4:** Progression-free survival in patients undergoing PCS by HRD score.

	Overall (*n* = 128)			HGS (*n* = 92)		
Characteristic	N	Hazard Ratio (95% CI)	*p*	N	Hazard Ratio (95% CI)	*p*
HRD score						
<42	53	Ref		35	Ref	
≥42	41	0.75 (0.47–1.21)	0.246	38	0.57 (0.34–0.95)	0.033
HRD score						
<33	45	Ref		27	Ref	
≥33	49	0.72 (0.45–1.16)	0.177	46	0.46 (0.27–0.77)	0.003
Any HRD (HRD score ≥42) ^1^						
No	42	Ref		26	Ref	
Yes	48	0.71 (0.44–1.16)	0.170	54	0.57 (0.33–0.98)	0.044
Any HRD (HRD score ≥33) ^2^						
No	36	Ref		20	Ref	
Yes	55	0.66 (0.41–1.07)	0.092	51	0.43 (0.24–0.75)	0.003

HGS = high-grade serous. HRD = homologous recombination deficiency. PCS = primary cytoreductive surgery. ^1^ Any HRD (HRD score ≥42) = germline or somatic BRCA1/2 mutation, germline non-BRCA1/2 homologous recombination deficiency mutation, BRCA1 promoter methylation (≥15), and/or HRD score ≥42). ^2^ Any HRD (HRD score ≥33) = germline or somatic BRCA1/2 mutation, germline non-BRCA1/2 homologous recombination deficiency mutation, BRCA1 promoter methylation (≥15), and/or HRD score ≥33).

**Table 5:** Multivariable model for overall and progression-free survival.

	Overall Survival			Progression-Free Survival		
Characteristic	N	Hazard Ratio (95% CI)	*p*	N	Hazard Ratio (95% CI)	*p*
Any BRCA1 mutation	67	0.76 (0.31–1.89)	0.557	67	0.63 (0.29–1.35)	0.233
Any BRCA2 mutation	84	0.19 (0.03–1.41)	0.105	84	0.51 (0.18–1.42)	0.194
Any BRCA1/2 mutation	68	0.44 (0.19–1.02)	0.054	68	0.47 (0.24–0.92)	0.028
gNon-BRCA HR mutation	62	0.93 (0.26–3.34)	0.908	62	1.15 (0.33–4.09)	0.825
HRD score ≥42	78	0.69 (0.36–1.30)	0.250	77	0.72 (0.42–1.24)	0.233
HRD score ≥33	78	0.43 (0.23–0.81)	0.009	77	0.62 (0.36–1.06)	0.078
BRCA1p methylation ≥15	70	1.41 (0.49–4.00)	0.522	69	1.11 (0.38–3.23)	0.841
Any HRD (HRD score ≥42) ^1^	73	0.68 (0.34–1.33)	0.260	71	0.70 (0.38–1.27)	0.240
Any HRD (HRD score ≥33) ^2^	72	0.45 (0.23–0.89)	0.022	72	0.60 (0.33–1.08)	0.087

gNon-BRCA HR mutation = germline non-BRCA1/2 homologous recombination deficiency mutation. HR = homologous recombination. HR = homologous recombination deficiency. BRCA1p = BRCA1 promoter. Multivariable model adjusted for age, stage, histology, and debulking status; low-grade histologic subtypes were excluded from the models. ^1^ Any HRD (HRD score ≥42) = germline or somatic BRCA1/2 mutation, germline non-BRCA1/2 homologous recombination deficiency mutation, BRCA1 promoter methylation (≥15), and/or HRD score ≥42). ^2^ Any HRD (HRD score ≥33) = germline or somatic BRCA1/2 mutation, germline non-BRCA1/2 homologous recombination deficiency mutation, BRCA1 promoter methylation (≥15), and/or HRD score ≥33).

## Data Availability

A portion of de-identified data presented in this study may be made available on request from the corresponding author. The data was stored in a secure database using REDCap (Research Electronic Data Capture) electronic data capture tools hosted at the University of Texas MD Anderson Cancer Center.

## Appendix A

**Table A1:** Microsatellite instability and tumor mutational burden testing in patients undergoing PCS.

Tumor Testing	*n* (%)
MSI status (*n* = 110)	
MSS	106 (96.4%)
MSI	1 (0.9%)
Failed testing	3 (2.7%)
TMB (*n* = 94)	
Median (range)	1.82 (0–30.4)
Low	93 (98.9%)
High	1 (1.1%)

MSI = microsatellite instability. MSS = microsatellite stable. PCS = primary cytoreductive surgery. TMB = tumor mutational burden.

**Table A2:** Overall survival in patients undergoing PCS.

Characteristic	N	Hazard Ratio (95% CI)	*p*	N	Hazard Ratio (95% CI)	*p*
Age						
<65 ≥65	76 52	Ref 2.57 (1.54–4.27)	<0.001	50 42	Ref 2.88 (1.64–5.07)	<0.001
Stage						
I/II	25	Ref		14	Ref	
III/IV	95	9.58 (2.33–39.37)	0.002	73	6.44 (1.56–26.58)	0.010
Residual disease after PCS						
Optimal	103	Ref		74	Ref	
Suboptimal (>1 cm)	9	4.16 (1.93–8.95)	<0.001	7	3.57 (1.47–8.65)	0.005
Any BRCA1 mutation						
No	69	Ref		44	Ref	
Yes	21	0.56 (0.25–1.26)	0.160	19	0.35 (0.15–0.85)	0.020
Any BRCA2 mutation						
No	108	Ref		74	Ref	
Yes	10	0.15 (0.02–1.08)	0.059	10	0.11 (0.01–0.79)	0.029
Any BRCA1/2 mutation						
No	62	Ref		37	Ref	
Yes	31	0.36 (0.17–0.77)	0.008	29	0.19 (0.08–0.44)	<0.001
gNon-BRCA HR mutation						
Negative	80	Ref		53	Ref	
Positive	5	1.97 (0.70–5.59)	0.200	4	1.95 (0.68–5.58)	0.214
BRCA1 promoter methylation						
<15	86	Ref		60	Ref	
≥15	7	1.14 (0.41–3.17)	0.804	6	0.68 (0.21–2.20)	0.516

gNon-BRCA HR mutation = germline non-BRCA1/2 homologous recombination mutation. HGS = high-grade serous. HR = homologous recombination. HRD = homologous recombination deficiency. PCS = primary cytoreductive surgery.

**Table A3:** Progression-free survival in patients undergoing PCS.

	Overall (*n* = 128)			HGS (*n* = 92)		
Characteristic	N	Hazard Ratio (95% CI)	*p*	N	Hazard Ratio (95% CI)	*p*
Age						
<65 ≥65	75 52	Ref 1.87 (1.22–2.86)	0.004	49 42	Ref 1.82 (1.14–2.91)	0.012
Stage						
I/II	25	Ref		14	Ref	
III/IV	94	2.98 (1.53–5.81)	0.001	72	2.17 (1.04–4.29)	0.038
Residual disease after PCS						
Optimal	102	Ref		73	Ref	
Suboptimal (>1 cm)	9	4.15 (1.97–8.72)	<0.001	7	3.10 (1.35–7.08)	0.007
Any BRCA1 mutation						
No	69	Ref		44	Ref	
Yes	21	0.70 (0.38–1.29)	0.254	19	0.47 (0.24–0.90)	0.023
Any BRCA2 mutation						
No	108	Ref		74	Ref	
Yes	10	0.51 (0.20–1.26)	0.143	10	0.35 (0.14–0.87)	0.025
Any BRCA1/2 mutation						
No	62	Ref		37	Ref	
Yes	31	0.56 (0.33–0.98)	0.041	29	0.30 (0.16–0.54)	<0.001
gNon-BRCA HR mutation						
Negative	80	Ref		53	Ref	
Positive	5	1.85 (0.67–5.11)	0.237	4	2.99 (1.06–8.47)	0.039
BRCA1 promoter methylation						
<15	85	Ref		59	Ref	
≥15	7	0.78 (0.28–2.15)	0.633	6	0.49 (0.15–1.57)	0.229

gNon-BRCA HR mutation = germline non-BRCA1/2 homologous recombination deficiency mutation. HGS = high-grade serous. HR = homologous recombination. HRD = homologous recombination deficiency. PCS = primary cytoreductive surgery.
